# Organ recovery from deceased donors with prior COVID‐19: A case series

**DOI:** 10.1111/tid.13503

**Published:** 2020-12-01

**Authors:** Nikole A. Neidlinger, Jeannina A. Smith, Anthony M. D’Alessandro, David Roe, Tim E. Taber, Marcus R. Pereira, Amy L. Friedman

**Affiliations:** ^1^ University of Wisconsin Organ and Tissue Donation Program Madison WI USA; ^2^ Department of Medicine University of Wisconsin Madison WI USA; ^3^ Indiana Donor Network Indianapolis IN USA; ^4^ Department of Medicine Columbia University College of Physicians New York NY USA; ^5^ LiveOnNY New York NY USA

**Keywords:** Coronavirus, COVID‐19, deceased donor, donor‐derived infection, SARS‐CoV‐2, transmission

## Abstract

Although guidance documents have been published regarding organ donation from individuals with a prior history of COVID‐19 infection, no data exist regarding successful recovery and transplantation from deceased donors with a history of or positive testing suggesting a prior SARS‐CoV‐2 infection. Here, we report a case series of six deceased donors with a history of COVID‐19 from whom 13 organs were recovered and transplanted through several of the nation's organ procurement organizations (OPOs). In addition, at least two potential donors were authorized for donation but with no organs were successfully allocated and did not proceed to recovery. No transmission of SARS‐CoV‐2 was reported from the six donors to recipients, procurement teams, or hospital personnel. Although more studies are needed, organ donation from deceased donors who have recovered from COVID‐19 should be considered.

**Abbreviations**
BAL: bronchoalveolar lavage
COVID‐19: Coronavirus disease 2019
KDPI: kidney donor profile index
NAT: nucleic acid testing
NP: nasopharyngeal
OPO: Organ Procurement Organization
OPTN: Organ Procurement and Transplantation Network
PCR: polymerase chain reaction
SARS‐CoV‐2: Severe Acute Respiratory Syndrome Coronavirus 2
UNOS: United Network for Organ Sharing

## 1 INTRODUCTION

The recognition and subsequent declaration of the COVID‐19 as a pandemic has dramatically impacted the landscape of organ donation and transplantation in the United States. Some of the earliest publications on the topic highlighted the importance of developing protocols and screening modalities for potential organ donors, based on lessons from prior pandemics, and recommended against recovering organs from a donor with suspected or confirmed COVID‐19, citing risks not only to recipients but also health care and procuring teams.^1^, ^2^ Screening of potential organ donors in the United States began in March utilizing both clinical and epidemiologic factors, as laboratories worked to develop specific nucleic acid (NAT) testing. Organ Procurement Organizations (OPOs) followed other countries and began universal testing all potential organ donors given the risk of pre‐symptomatic or asymptomatic infection.^3^ A report from the Organ Procurement and Transplantation Network (OPTN) identified that 100% of deceased donors underwent NAT testing starting on April 21, 2020.^4^ Organ authorization and recovery rates dropped by 11 and 17%, respectively, over the ensuing 90‐day period from March to May, as social distancing reduced hospital admissions and traumatic deaths.^5^ It was universally accepted that any donor with a current COVID‐19 infection would be excluded from donating any organ. However, as transplant activity resumed in the summer months of 2020, OPOs predictably began to receive referrals for organ donation from patients with evidence of prior SARS‐CoV‐2 infection. As no data exist regarding the safety of such patients as organ donors, the purpose of this study is to describe our experience regarding organ donors with evidence or history of prior SARS‐CoV‐2 infection.

## 2 METHODS

There are 58 OPOs within the United States and Puerto Rico that are responsible for all aspects of deceased donor recovery including evaluation, screening, clinical management, allocation, and recovery. The Medical Directors for all 58 OPO’s were approached by email on 7/31/2020 regarding experience in their donation service area with deceased donors with a history of COVID‐19 in an effort to understand the nation's experience, if any. The OPO’s were asked two questions: 1) are you currently considering deceased donors with a history of COVID? 2) Have you recovered and transplanted organs from these donors? Information was collected voluntarily, with no compensation. Twenty‐eight OPO’s (48%) responded; two had unsuccessful attempts with such donors, the experience of the other three OPO’s is described here. The remaining 23 cited no experience.

Donors and families provide authorization for de‐identified data and relevant medical information to be collected and reported in order to facilitate quality improvement and patient safety in donation and transplantation. Collection of relevant information on potential disease transmission is a requirement of the OPO and de‐identified recipient data regarding any such transmission were collected by the OPO. No identifiable patient or donor information was shared.

### 2.1 Case 1

A 32‐year‐old man was declared neurologically dead in July 2020 following a drug overdose. The donor had a Kidney Donor Profile Index (KDPI) of 10%, and excellent organ function. A standard screening nasopharyngeal (NP) swab for SARS‐CoV‐2 Polymerase Chain Reaction (PCR) was negative. During allocation, it was discovered that the donor was diagnosed with COVID‐19 infection 14 weeks prior, after presenting with cough, fatigue, watery eyes, and diarrhea, and had a positive NP swab at that time. He was never hospitalized and recovered at home. This had not been detected on the initial DRAI, which inquired about exposures in the prior 28 days. The donor underwent a second PCR from bronchoalveolar lavage (BAL) fluid, which was negative. The heart, kidneys, and liver were accepted and transplanted. The donor's archived serum was found to be positive for SARS‐CoV‐2 IgG antibody post recovery. The liver recipient had no evidence of transmission at hospital discharge. The heart recipient had a smooth course and negative SARS‐CoV‐2 PCR 6 weeks post‐transplant. The left kidney recipient had delayed graft function but has since recovered at home with normal kidney function and two negative SARS‐CoV‐2 PCR. The right kidney recipient went home with no delayed graft function, and has had no further SARS‐CoV‐2 testing or signs of transmission.

### 2.2 Case 2

A 21‐year‐old woman presented with hemodynamic instability following blunt trauma in July 2020. She was coagulopathic and received massive transfusions. A routine admission NP swab for SARS‐CoV‐2 was negative. Chart review revealed that the patient had complained of fever and respiratory symptoms 38 days prior to the trauma admission, was diagnosed with COVID‐19 by NP swab at that time, and recovered uneventfully at home. Despite normal admission creatinine of 0.93 mg/dL, she developed non‐oliguric acute kidney injury, with a terminal creatinine of 4.5mg/dL. Her KDPI was 27%. After stabilization, she was declared dead by neurologic criteria. A second SARS‐CoV‐2 PCR was reported as negative on tracheal aspirate. The heart, liver, and kidneys were accepted for transplantation. In the donor OR, the kidneys were found to have acute tubular necrosis and severe thrombotic microangiopathy with intracapillary fibrin thrombi. The kidneys were discarded, and the liver and heart were successfully transplanted. Post‐transplant, hemodiluted donor serum tested negative for SARS‐CoV‐2 IgG antibody. The liver recipient has recovered uneventfully and was discharged home, with no sign of transmission at 21 days post‐transplant and a negative SARS‐CoV‐2 IgG antibody test. The heart recipient was discharged home and has no sign of transmission.

### 2.3 Case 3

A 54‐year‐old with end‐stage kidney disease, hypertension, diabetes, prior stroke, and multiple previous amputations was admitted following a cardiac arrest during routine hemodialysis. The patient was a nursing home resident who had been tested for coronavirus 3 months prior to this event and was negative at that time by NP swab. On this admission, screening NP swab was negative for coronavirus. The patient was declared neurologically dead and deemed a suitable donor, with no known history of coronavirus. During donor evaluation 3 days later, a second NP PCR was positive for SARS‐CoV‐2. On that same day, both stool and BAL PCRs were negative. Antibody testing showed that the donor was SARS‐CoV‐2‐IgM negative but IgG positive. The next day, the liver was recovered and transplanted. No other organ was deemed suitable for transplant. The liver recipient died of multisystem organ failure 1 month post‐transplant and never left the hospital. SARS‐CoV‐2 PCR testing was done pre‐mortem three times in the post‐operative period and was negative on post‐transplant days 1, 3, and 23.

### 2.4 Case 4

A 46‐year‐old with a history of hypertension and obesity was declared dead by neurological criteria after suffering a stroke. Chart review found that the patient had been diagnosed with an asymptomatic SARS‐CoV‐2 infection 48 days prior by NP swab. Following the stroke, the admission SARS‐CoV‐2 NP swab was negative but IgG level was positive at 25.9 AU/mL. Repeat PCR testing found a second negative NP swab and a negative BAL. The liver was transplanted, the recipient tested negative for SARS‐CoV‐2 post‐transplant and was still recovering in the hospital 2 weeks later.

### 2.5 Case 5

A 52‐year‐old with end‐stage liver disease suffered a stroke and neurologic death. The patient had been admitted to a hospital three times between April 9 and June 29 with complications of cirrhosis. On all three admissions, COVID‐19 was listed as a confounding diagnosis. On another hospitalization in July 2020 related to cirrhosis, the patient's NP swab was negative and two SARS‐CoV‐2 IgGs were positive with a level of 26.7 AU/mL. After being declared dead by neurologic criteria in the week prior to recovery, the donor had negative SARS‐CoV‐2 PCR testing in two SARS‐CoV‐2 NP swabs and one BAL. The kidneys were recovered and transplanted. Both recipients recovered uneventfully and left the hospital. The right kidney recipient had a negative SARS‐CoV‐2 PCR 18 days post‐transplant. The left kidney recipient has not been tested. Neither recipient has any sign of transmission at 4 weeks post‐transplant.

### 2.6 Case 6

A 22‐year‐old with a history of epilepsy suffered cardiac arrest caused by seizure and was declared dead by neurologic criteria. Admission SARS‐CoV‐2 NP swab was negative, and the donor had no known history of coronavirus infection. On hospital day 2, a SARS‐CoV‐2 PCR from the BAL was negative. The heart, liver, and kidneys were allocated and during their recovery on hospital day 3, a SARS‐CoV‐2 stool PCR came back positive but a repeat NP swab was negative. The heart recipient was already in the operating room when the tests resulted. The liver transplant was aborted, but heart and kidneys were successfully recovered and transplanted. The heart recipient is recovering in the hospital with a negative SARS‐CoV‐2 NP PCR and no sign of transmission. The kidney recipients have both recovered uneventfully and been discharged home with negative SARS‐CoV‐2 PCR testing and show no sign of transmission at 30 days post‐transplant.

In addition to the donor and recipient information, these six recoveries involved nine procurement teams, including five teams local to the donation service area and four visiting teams. No transmission to any member of the procurement, operating room, or hospital teams was reported. The demographic factors and testing data for these donors and recipients are summarized in Table 1.

**Table 1:** Factors and testing data in six deceased donors with suspected or documented history of COVID‐19 and the 13 recipients of their organs

	Case 1	Case 2	Case 3	Case 4	Case 5	Case 6
Donor Age	32	21	54	46	52	22
Donor COVID‐19 illness	Diagnosed By PCR Recovered at home	Diagnosed by PCR Recovered at home	Unknown when infection occurred	Diagnosed by PCR Recovered at home	Diagnosed by PCR Hospitalized	Unknown
Time from infection to donation	14 weeks	38 days	Unknown	48 days	4 months	Unknown
SARS‐CoV‐2 donor testing on terminal admission	NP PCR negative BAL PCR negative	NP PCR negative Tracheal Aspirate PCR negative	NP PCR negative x1 NP PCR positive x1 Stool PCR negative BAL PCR negative IgM negative IgG positive	NP PCR negative x2 BAL PCR negative IgG positive	NP PCR negative BAL PCR negative IgG positive	NP PCR negative BAL PCR negative Stool PCR positive NP PCR negative
Organs Transplanted	Heart, Liver, Kidneys	Heart, Liver	Liver	Liver	Kidneys	Heart, Kidneys
Post recovery SARS‐CoV‐2 testing in donor and recipients	Donor‐IgG positive Heart‐NP PCR negative Liver—none Left kidney‐NP PCR negative x2 Right kidney—none	Donor‐IgG negative Heart—unknown Liver‐IgG negative and NP PCR negative	Liver‐NP PCR negative x3	Liver‐NP PCR negative	Right kidney‐NP PCR negative Left kidney—none	Heart‐NP PCR negative Left kidney‐ NP PCR negative Right kidney‐NP PCR negative
Recipient outcome	No known transmission No reported death	No known transmission No reported death	No known transmission Liver death MSOF	No known transmission No reported death	No known transmission No reported death	No known transmission No reported death

Abbreviations: BAL, bronchoalveolar lavage; IgG, Immunoglobulin G antibody; IgM, immunoglobulin M antibody; MSOF, multisystem organ failure; NP, nasopharyngeal; PCR, Polymerase Chain Reaction; SARS‐CoV‐2, Severe Acute Respiratory Syndrome Coronavirus 2.

## 3 DISCUSSION

Coronavirus was declared a pandemic on March 11, 2020, and changed organ and tissue donation dramatically. The calendar year 2019 saw a record‐breaking 11,870 deceased donors in the United States,^6^ representing nearly 40% increase from just 5 years prior. Prior to March 11, the nation was recovering more than 250 deceased donors per week, on pace again for a record year. After the announcement, organ recovery rates in the United States mimicked the decline previously seen in Italy and plummeted by more than 25%, as transplant hospitals recognized the risks of immunosuppression, resource utilization, and staff deployment in the face of an overwhelmed medical system.^7^, ^8^, ^9^ By late April, these rates slowly began to increase as centers and OPOs began to navigate resource availability and risk‐benefit analysis. Operational changes for OPO’s included limited onsite presence at partner hospitals, telephone approaches for authorization, and increases in local recovery and centralized recovery centers.^2^ Authorization rates declined as did overall donation and transplantation rates.^5^, ^10^

Patients referred as potential organ donors undergo epidemiologic and clinical screening and any confirmed diagnosis of COVID‐19, whether current or in the prior 28 precludes donation.^3^ Laboratories developed rapid turnaround testing availability and OPO’s began universal screening of all consented donors, generally by NP swab, given provider exposure risks with other methods.^11^ Later, it has been common to also perform BAL testing. However, even in the face of negative testing, there have been continued concerns about the safety of donation. In particular, there have been concerns that the screening tests may be insufficiently sensitive, that donors may still be in the asymptomatic phase of infection before virus is detectable, and concerns exist regarding nosocomial spread during donor workup. In the epicenter of Wuhan, deceased donors are quarantined in intensive care for at least 7 days during the workup period and must undergo two negative NAT and antibody tests prior to donating.^12^

Organ donors with respiratory viruses such as influenza and the pandemic H1N1 2009 influenza virus have had organs successfully recovered and transplanted, however, the systemic nature of COVID‐19 manifestations raised questions as to whether SARS‐CoV‐2 involvement of organs and tissues beyond the respiratory tract would complicate donation of non‐lung organs from such donors.^13^, ^14^ Early studies from the virus epicenter described the initial cluster of 41 known COVID‐19 patients, only 15% had viremia.^15^ Another study examined more than 1000 tissue specimens from 200 COVID‐19 patients and found that while 29% had virus in the GI tract, very few had detectable virus in blood or urine.^16^ However, autopsy studies in patients who died of COVID‐19 found evidence of systemic infection; more than 60% of decedents had virus present in renal and cardiac tissue.^17^

Three living donors with a history of COVID‐19 have been reported, with one potential transmission. A 21‐year‐old man received apheresis platelets from a presumed healthy donor, the donor was diagnosed with COVID‐19 3 days after the blood donation; 1 day following the transfusion, and presumably had an asymptomatic infection at the time of donation. The recipient was immunosuppressed and had no sign of transmission in three screening tests done over the subsequent 2 weeks.^18^ Also, a 28‐year‐old woman was admitted as a living liver donor with a mild febrile illness. The following day she donated a liver segment and 2 days later was notified about a recent COVID‐19 exposure. On post‐operative day 3, the living donor was confirmed SARS‐CoV‐2 positive. The recipient had no sign of donor‐derived SARS‐CoV‐2 infection by testing or clinical suspicion for 69 days post‐operatively, or did any healthcare personnel develop infection.^19^ One reported transmission occurred when an infant received a liver graft from her mother; the donor was subsequently found to be SARS‐CoV‐2 positive on post‐operative day 2. The infant developed a febrile illness and hepatitis and was diagnosed with COVID‐19 2 days later, but had not required intubation as of 2 weeks post‐transplant.^20^ In this case, since the donor was the child's mother, it is unclear if the transmission was through the donor organ or via exposure to respiratory droplets in the setting of close contact between mother and child. There are no reports of transmission of COVID‐19 from deceased donors to date, leading some authors to advocate for consideration of SARS‐Cov2‐positive donors.^21^

To our knowledge no data exist regarding organs transplanted from deceased donors with a past history of COVID‐19 or positive PCR. Guidance documents have suggested a “wait time” of 28 days prior to consideration and universal screening questionnaires have cited this same time frame when obtaining the decedent's medical history. Inquiry to medical directors of the nation's 58 OPO’s revealed that more than half had no experience with such donors, or were unable to place organs from these donors for transplantation. In the current series, one of the six decedents required hospital treatment of their coronavirus infection, while the others were asymptomatic or recovered as outpatients, therefore, subjective information regarding their disease severity is subjective as reported by their family and legal next of kin surrogates. Time interval from documented infection to donation ranged from 38 days to more than 90 days. None of the donors in this series had lungs or pancreas recovered or transplanted. Two of the six donors had no documented history of COVID‐19 but had discordant testing during donor workup including a positive PCR test in the setting of several other negative tests.

It has been observed that NP and BAL PCR testing for SARS‐COV‐2 can remain positive or be intermittently positive for weeks after COVID‐19 infection. Initially, it was unknown if these patients had virus capable of replication, or if the PCR was detecting non‐replicative competent viral fragments. It was therefore unknown if they should remain in isolation. The PCR cycle threshold was thought to be helpful in this determination, but for the cases here noted, with reversion to positive PCR testing, the cycle threshold values were not available. More recently, the longest interval of detection of replication‐competent virus in nasopharyngeal swabs was 20 days.^22^ On the basis of this and similar studies at the CDC, the isolation requirements for patients with a history of NP PCR positivity changed, and is based on time from first positive test or onset of symptoms, and re‐testing to “clearance” is no longer encouraged. That being said, it is currently unknown if replicative‐competent virus might persist longer in tissues which could be transmitted by organ transplantation.

The current series is limited in that it may under‐represent the true number of deceased donors with history of COVID given its design as a retrospective case series and reliance on self‐reporting. We urge all OPO’s to report any donor, even if they do not currently test positive but has a history of COVID‐19, to the OPTN via the patient safety portal. SARS‐CoV‐2 has been included as a pathogen of special interest by the Disease Transmission Advisory Committee (DTAC) and it is critical to systematically analyze these events in the interest of patient safety. An OPTN report from July 1 indicated that there is at least one additional pediatric donor who had a positive SARS‐CoV‐2 IgG antibody test in May 2020. The donor had a negative PCR and the antibody result was attributed to the child's birth mother having been diagnosed with COVID‐19 during pregnancy. There were no other donors with any positive COVID‐19 test reported to OPTN as of July 1.^4^ All three of the donors in this series occurred after July 1. We acknowledge that follow‐up data regarding potential transmission is incomplete and may be underestimated, and that this report is limited in its ability to draw meaningful conclusions regarding the safety or transmission rates from deceased donors with a history of COVID‐19. We acknowledge the difficulty in excluding COVID‐19 transmission in recipients, as no universally agreed upon post‐transplant testing protocol exists, and any antibody testing would need to be considered in light of induction and maintenance immunosuppression. Nonetheless, it represents the first reported series of such donors and highlights the need for ongoing evaluation of donation in the setting of recovered COVID‐19 infection. More than 5.5 million Americans have recovered from a coronavirus infection, inevitably some of these patients will die of unrelated illnesses and present as potential organ and tissue donors. We advocate for the donation and transplantation community to carefully weigh the risks and benefits rather than immediately disqualify organs from donors with this history. This series suggests that there may be benefit when the death is unrelated to coronavirus, does not occur at the index admission, and when COVID‐19 infection was at least 28 days before donation.

## CONFLICT OF INTEREST

The authors declare no conflicts of interest.

## AUTHOR CONTRIBUTIONS

NN and AF designed and directed the project; NN, AD, TT, DR, MP, and AF collected and contributed specific clinical data on the patient series; JS and MP provided critical ID analysis and interpretation of the data; NN, JS, and AF drafted the paper with critical input from all authors; and all seven authors approved the final version and agree to accountability for all aspects of the work.

## References

[1] Micheals MG , La Hoz RM , Danziger‐Isakov L , et al. Coronavirus disease: implications of emerging infections for transplantation. Am J Transplant. 2019;20(7):1768‐1772. 10.1111/ajt.15832 PMC9800450 32090448

[2] Friedman AL , Delli Carpini KW , Ezzell C , Irving H . There are no best practices in a pandemic: organ donation within the COVID‐19 epicenter. Am J Transplant. 2020;20(11):3089‐3093. 10.1111/ajt.16157 32568471 PMC7361464

[3] Kumar D , Manuel O , Natori Y , et al. COVID‐19: a global transplant perspective on successfully navigating a pandemic. Am J Transplant. 2020;20(7):1773‐1779. 32202064 10.1111/ajt.15876 PMC7228301

[4] Cartwright L , Wilk A , Taranto S , et al. Summary of COVID‐19 Emergency Policy and IT Changes. Descriptive Data Request for the OPTN Executive Committee. Published July 1, 2020. https://optn.transplant.hrsa.gov/media/3985/data_report_executivecmte_covid_20200701_rpt3.pdf

[5] Ahmed O , Brockmeier D , Lee K , Chapman WC , Doyle MBM . Organ donation during the COVID‐19 pandemic. Am J Transplant. 2020;20(11):3081‐3088. 10.1111/ajt.16199 32659028 PMC7404840

[6] Organ Procurement and Transplantation Network National Data. https://optn.transplant.hrsa.gov/data/view‐data‐reports/national‐data/. Accessed on September 1, 2020.

[7] Angelico R , Trapani S , Manzia TM , Lombardini L , Tisone G , Cardillo M . The COVID‐19 outbreak in Italy: initial implications for organ transplantation programs. Am J Transplant. 2020;20(7):1780‐1784. 10.1111/ajt.15904 32243677 PMC9800493

[8] Gori A , Dondossola D , Antonelli B , et al. Coronavirus disease 2019 and transplantation: a view from the inside. Am J Transplant. 2020;20(7):1939‐1940. 10.1111/ajt.15853 32181969 PMC9800453

[9] Halazun KJ , Rosenblatt R . Lest we forget. Am J Transplant. 2020;20:1785‐1786. 10.1111/ajt.15888 32233055 PMC9800466

[10] Collins K , Doyle M . Revisiting the OPO‐based Organ Procurement Center (OPC) in the COVID era. Am J Transplant. 2020. 10.1111/ajt.16124 32503083

[11] The American Society of Transplant Surgeons . Organ Retrieval for Transplantation in the COVID‐19 Era. https://asts.org/advocacy/covid‐19‐resources/asts‐covid‐19‐strike‐force/asts‐covid‐19‐strike‐force‐organ‐retrieval‐guidance#.X1vDWmhKhaR. Published March 27, 2020

[12] Shi H , Xu J , Li X , et al. First organ donation in Wuhan after ending of the coronavirus lockdown. Transpl Int. 2020;33:1149‐1150. 10.1111/tri.13658 32429004 PMC7280711

[13] Lattes R , Jacob N , de la Fuente J , Fragale G , Massari P . Pandemic influenza A/H1N1 and organ donation. Transpl Infect Dis. 2010;12(2):169‐172. 10.1111/j.1399-3062.2010.00494.x 20180928

[14] Green M , Covington S , Taranto S , et al. Donor‐derived transmission events in 2013: a report of the Organ Procurement Transplant Network Ad Hoc Disease Transmission Advisory Committee. Transplantation. 2015;99(2):282‐287. 10.1097/TP.0000000000000584 25594557

[15] Huang C , Wang Y , Li X , et al. Clinical Features of patients infected with 2019 novel coronavirus in Wuhan, China. Lancet. 2020;395(10223):497‐506. 10.1016/S0140-6736(20)30183-5 31986264 PMC7159299

[16] Wang W , Xu Y , Gao R , et al. Detection of SARS‐CoV‐2 in Different Types of Clinical Specimens. JAMA. 2020;323(18):1843‐1844. 10.1001/jama.2020.3786 32159775 PMC7066521

[17] Puelles V , Lutgehetmann M , Lindenmeyer M , et al. Multiorgan and Renal Tropism of SARS‐CoV2. NEJM. 2020;383:590‐592. 32402155 10.1056/NEJMc2011400 PMC7240771

[18] Cho HJ , Koo JW , Roh SK , et al. COVID‐19 transmission and blood transfusion: A case report. J Infect Public Health. 2020;13(11):1678‐1679. 32405329 10.1016/j.jiph.2020.05.001 PMC7218366

[19] Hong H‐L , Kim S‐H , Choi DL , Kwon HH . A case of coronavirus disease 2019–infected liver transplant donor. Am J Transplant. 2020;23:1‐4. 10.1111/ajt.15997 PMC7272801 32400013

[20] Lagana S , De Michele S , Lee M , et al. COVID‐19–associated hepatitis complicating recent living donor liver transplantation. Arch Pathol Lab Med. 2020;144(8):929‐932. 10.5858/arpa.2020-0186-SA 32302212

[21] Kates OS , Fisher CE , Rakita RM , Reyes JD , Limaye AP . Emerging evidence to support not always “just saying no” to SARS‐CoV‐2 positive donors. Am J Transplant. 2020;20(11):3261‐3262. 10.1111/ajt.16119 32502313 PMC7300931

[22] Rhee C , Kanjilal S , Baker M , Klompas M . Duration of SARS‐CoV‐2 Infectivity: when is it safe to discontinue isolation? Clin Infect Dis, ciaa1249, 10.1093/cid/ciaa1249 PMC7499497 33029620

